# In Situ Labeling and Distance Measurements of Membrane Proteins in *E. coli* Using Finland and OX063 Trityl Labels

**DOI:** 10.1002/chem.202004606

**Published:** 2021-01-14

**Authors:** Sophie Ketter, Aathira Gopinath, Olga Rogozhnikova, Dmitrii Trukhin, Victor M. Tormyshev, Elena G. Bagryanskaya, Benesh Joseph

**Affiliations:** ^1^ Institute of Biophysics Department of Physics Goethe University Frankfurt Max-von-Laue-Str. 1 60438 Frankfurt/Main Germany; ^2^ N. N. Vorozhtsov Novosibirsk Institute of Organic Chemistry SB RAS Pr. Lavrentieva 9 Novosibirsk 630090 Russia

**Keywords:** DEER or PELDOR, FTAM and OX063, membrane protein, spin labeling, β-barrel assembly machinery (BAM) complex

## Abstract

In situ investigation of membrane proteins is a challenging task. Previously we demonstrated that nitroxide labels combined with pulsed ESR spectroscopy is a promising tool for this purpose. However, the nitroxide labels suffer from poor stability, high background labeling, and low sensitivity. Here we show that Finland (FTAM) and OX063 based labels enable labeling of the cobalamin transporter BtuB and BamA, the central component of the β‐barrel assembly machinery (BAM) complex, in *E coli*. Compared to the methanethiosulfonate spin label (MTSL), trityl labels eliminated the background signals and enabled specific in situ labeling of the proteins with high efficiency. The OX063 labels show a long phase memory time (*T_M_*) of ≈5 μs. All the trityls enabled distance measurements between BtuB and an orthogonally labeled substrate with high selectivity and sensitivity down to a few μm concentration. Our data corroborate the BtuB and BamA conformations in the cellular environment of *E. coli*.

## Graphical abstract

**Observing membrane proteins in the cellular environment** is a difficult task and PELDOR/DEER spectroscopy is a versatile tool for this purpose. The nitroxide spin labels suffer from poor stability and sensitivity in cellular environments. Trityls are emerging as promising tags for in situ PELDOR/DEER. Here we show that FTAM and OX063 labels provide superior sensitivity and selectivity for distance measurements of membrane proteins in *E. coli*.

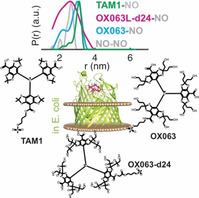

Membrane protein structures are often determined in a non‐native environment, which masks the effect of cellular conditions. While significant efforts are being made to observe soluble proteins in their native environments, in‐cell investigation of membrane proteins is still a challenging task. Pulsed electron‐electron double resonance (PELDOR or DEER) spectroscopy[^1^, ^2^] has emerged as a powerful tool for structural biology, especially of membrane proteins.[^3^, ^4^, ^5^, ^6^] Over the past years, we demonstrated that PELDOR/DEER spectroscopy can be used to observe the structure and conformational changes of membrane proteins in the native membranes and intact *E. coli*.[^7^, ^8^, ^9^] However, limited stability of the MTSL, background labeling, and a rather broad spectrum diminished the overall sensitivity. Reduction‐resistant nitroxide and Gd^3+^ labels were shown to possess significantly increased stability in cellular environments.[^4^, ^10^, ^11^, ^12^, ^13^]

Membrane proteins are expressed at a very low level (ranging between 10^2^–10^5^ copies per cell) and to observe them close to the native expression level, very sensitive spin labels and labeling strategies need to be developed. Also, several membrane proteins form homo‐ or heterooligomeric complexes^[14]^ and orthogonal labeling strategies would greatly facilitate their investigations. The carbon‐centered trityl radicals have attained much attention for ESR and also as an orthogonal tag with other spin labels.[^8^, ^15^, ^16^, ^17^, ^18^, ^19^, ^20^, ^21^, ^22^] Like the Gd^3+^‐based labels, they are stable in the reducing cellular environment and their narrow linewidth provides high sensitivity. Despite the long phase memory time (*T_M_*) at temperatures ≤50 K, the transverse relaxation time (*T_1_*) for trityls gets too long, which reduces the overall sensitivity. At temperatures ≥100 K, *T_1_* becomes more favorable, which allows faster data acquisition. Although this is accompanied with a drastic reduction of the *T_M_* at higher temperatures,[^23^, ^24^] PELDOR/DEER experiments using trityls have been performed even at ambient temperatures.[^16^, ^25^, ^26^] For soluble proteins, in‐cell Fe^II^‐trityl and trityl‐trityl distance measurements have been reported,[^17^, ^26^, ^27^, ^28^] while similar applications with membrane proteins are yet to be demonstrated.

The first‐generation Finland trityl (FTAM) based labels[^16^, ^25^, ^29^] displayed low water solubility and a tendency for aggregation. Also, attaching these labels to biomolecules lead to a drastic reduction of the *T_M_*.[^16^, ^19^, ^25^] To increase the solubility of the trityl labels, the FTAM core was replaced with more hydrophilic OX063 core, which not only reduced the aggregation but also significantly increased the *T_M_*.^[30]^ So far, PELDOR/DEER experiments using trityls were reported using the FTAM‐based labels and for the in‐cell experiments, labeled proteins were exogenously introduced into the cells. Thus, it is unknown how the solubility modification may affect the interaction of the labels with its surroundings or how the trityls would perform when a target protein is labeled directly in the cellular environment.

The three trityl labels we employed in the present study are shown in Figure 1. All of them are functionalized with a methanethiosulfonate group for specific reaction with an engineered cysteine in the target protein. TAM1 is a FTAM based label[^8^, ^31^] whereas the other two labels are based on the OX063 core and the OX063L‐d24 is the deuterated analog.^[30]^ Previously we had shown that TAM1 can be used to label the cobalamin transporter BtuB in isolated membranes (OM) and binding to the TEMPO‐labeled cobalamin (T‐CNCbl) was observed using PELDOR.^[8]^ BtuB transports vitamin B_12_ from the extracellular environments into the periplasm. This process is believed to be energized by the interaction of the BtuB Ton‐box with the TonB‐ExbB‐ExbD complex located in the inner membrane.^[32]^ Labeling BtuB with TAM1 in the membranes lead to severe aggregation as well as a significantly reduced *T_M_* (≈1.2 μs at 50 K). The decreased lipophilicity of the OX063 labels was shown to almost eliminate the aggregation and OX063L‐d24 labeled on human serum albumin (HSA) showed the longest *T_M_* (6.3 μs) yet obtained with any trityl label.^[30]^ So far, we employed BtuB for the ESR experiments in *E. coli*. Here we also used BamA, the central component of the β‐barrel assembly machinery complex (BAM) in Gram‐negative bacteria.^[33]^ BAM complex consists of BamA and 4 other interacting lipoproteins BamB‐E. It is responsible for the folding and insertion of the majority of outer membrane proteins in Gram‐negative bacteria and is one of the most sought‐after targets for novel antibiotics.[^34^, ^35^, ^36^]

**Figure 1:**
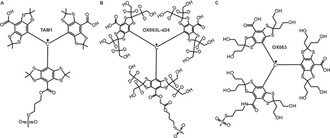
Structure of the trityl radicals used in the present study. a) The FTAM‐based TAM1, b) deuterated OX063L‐d24, and c) non‐deuterated OX063. All the labels are methanethiosulfonate‐functionalized for selective reaction with an engineered cysteine in the target protein. TAM1 and OX063 possess a similar linker and the OX063L‐d24 has a longer diester linker.

We overexpressed BtuB carrying the T188C mutation on the second extracellular loop and performed labeling in *E. coli* using the three trityl labels (Figure 2 a–b). Spin labeling was performed at a cell density corresponding to OD_600_=5 using 100 μm spin labels at room temperature for 15 min. Surprisingly, trityl labels nearly eliminated the background labeling and enabled specific labeling of the T188C mutant. Such a difference between the WT and the 188C mutant could not be observed in the OM due to aggregation and PELDOR/DEER experiment was facilitated through a fast and selective relaxation of the TAM1 clusters.^[8]^ MTSL does not form aggregates, but it consistently produced a significant amount of non‐specific labeling.^[9]^ Though such stochastic labeling does not interfere with PELDOR/DEER experiments, in effect it decreases the overall sensitivity through reducing the modulation depth. The non‐selective porins in the outer membrane exclude molecules with a size >600 Da. Owing to their larger size and the unique properties of the outer membrane, trityls might be excluded from entering the membranes or the porins, and thereby suppress the non‐specific labeling.

**Figure 2:**
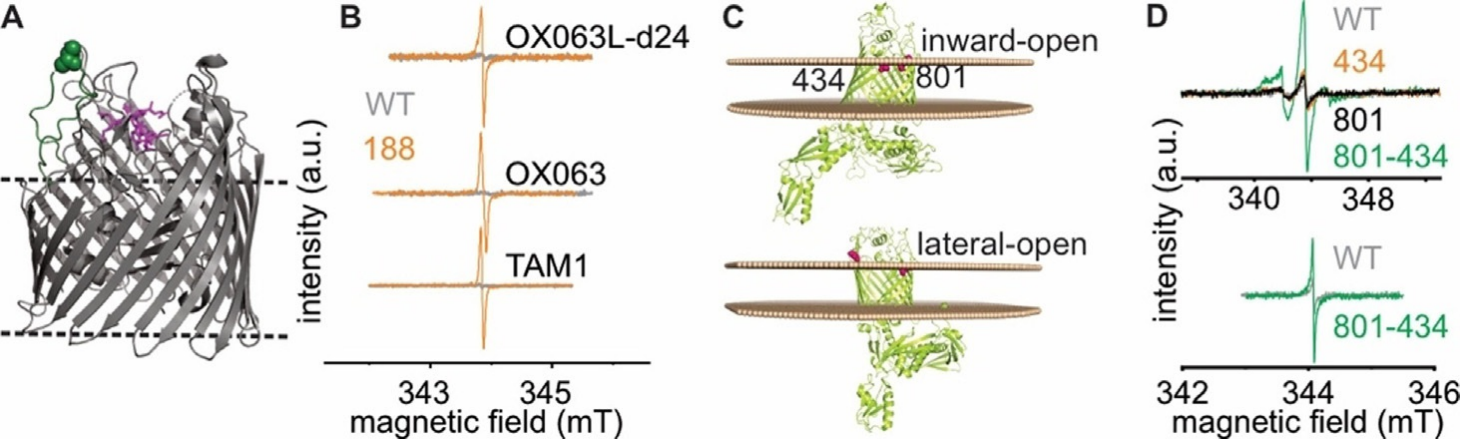
In situ labeling of BtuB and BamA using FTAM, OX063, and MTSL labels in *E. coli*. a) The structure of BtuB with bound cyanocobalamin (1NQH) with the second extracellular loop highlighted in green and the atoms of the labeled position 188C shown as spheres. The bound cyanocobalamin is shown using stick representation in magenta. b) Room temperature continuous wave (RT CW) ESR spectra for BtuB 188C or the WT protein labelled with different trityl labels as indicated. The derivative spectrum for the OX063L‐d24 label is visibly asymmetric, the reason for which is not clear at present. c) BamA inward‐open (5D0O) or lateral‐open (5LJO) structures with the membrane bilayer as predicted by the Orientations of Proteins in Membranes (OPM) server. In the former conformation, the labeled positions (434 and 801) are located inside the bilayer, and in the latter conformation, the position 434 is selectively exposed out of the membrane bilayer. The BamB‐E subunits are not shown d) RT CW ESR spectra for BamA 434C, 801C, and 801C‐434C mutants using MTSL (top) or using OX063L‐d24 for 801C‐434C (bottom).

BtuB labeled with the trityls revealed a narrow spectrum and gave a spin concentration in the range of 4 to 8 μm (Table 1). The modulation depths for the PELDOR/DEER data revealed a labeling efficiency of about 70 % for TAM1, 50 % for OX063, and 60 % for OX063L‐d24 (see the PELDOR/DEER section and Figure 4). Overall, this is comparable with the labeling efficiency achieved for MTSL (≈60 %).

**Table 1:** Spin concentrations and phase memory times (*T_M_*) for the FTAM and OX03 labels in *E. coli* overexpressing BtuB WT or the 188C mutant.

Spin label	WT (μm)	188C (μm)	*T_M_* 50 K (μs)	*T_M_* 100 K (μs)
OX063L‐d24	b.d.^[a]^	4.0±0.8^[b]^	4.7±0.3^[c]^	4.3±0.2
OX063	b.d.	5.0±1.0	5.1±0.2	4.7±0.2
TAM1	b.d.	8.0±1.6	2.9±0.1	2.9±0.1
T‐CNCbl	–	–	5.2±0.2	2.2±0.3

[a] b.d., below detection. [b] a maximum of 20 % error is estimated for the spin quantification. [c] Values are shown as mean±S.D. The S.D. was calculated for the values corresponding to signal to noise. For comparison, the *T_M_* values for the T‐CNCbl, which occupies the binding pocket inside BtuB is shown.

To further explore the performance of the trityls, we monitored their stability in *E. coli* suspension (at OD_600_=5). Trityls were shown to be very stable against biological redox agents.^[10]^ Interestingly, both the trityls and the MTSL showed unusual kinetics of their stability. For the trityls, the spin concentration increased during the first 1000 s, then declined suddenly, and followed with a rather stable phase with little or no loss of signal intensity (Figure 3 a and Figure S1). At OD_600_=0.7, this pattern disappeared and the trityls remained very stable. We conclude that despite their lower lipophilicity, even OX063 labels aggregate at higher cell densities. This led to an increase in the signal intensity, possibly due to the accumulation of trityl aggregates into the active resonator volume (from the suspension above) and or due to relaxation enhancement. At some point in the time course, these aggregates might become very large and move out of the resonator volume towards the bottom of the sample tube, leading to a sudden decrease in the observed spin concentration. Similar behavior has been reported previously for both FTAM and OX063, forming larger aggregates or even fibrils of trityls in solution.[^19^, ^37^] Despite this tendency, CW or pulsed ESR experiments did not reveal the presence of aggregates in the trityl‐labeled cell suspension (Figure 2 b, d, and 3d). Previously, we showed that such an aggregation in the native membranes lead to a significant line broadening in both RT CW ESR and echo‐detected field‐swept spectra (FS).^[8]^ With *E. coli* cells, the rigorous washing steps following spin labeling gives suitable conditions for a nearly complete dissolution of the aggregates, and huge line broadening was present when washing was insufficient.

**Figure 3:**
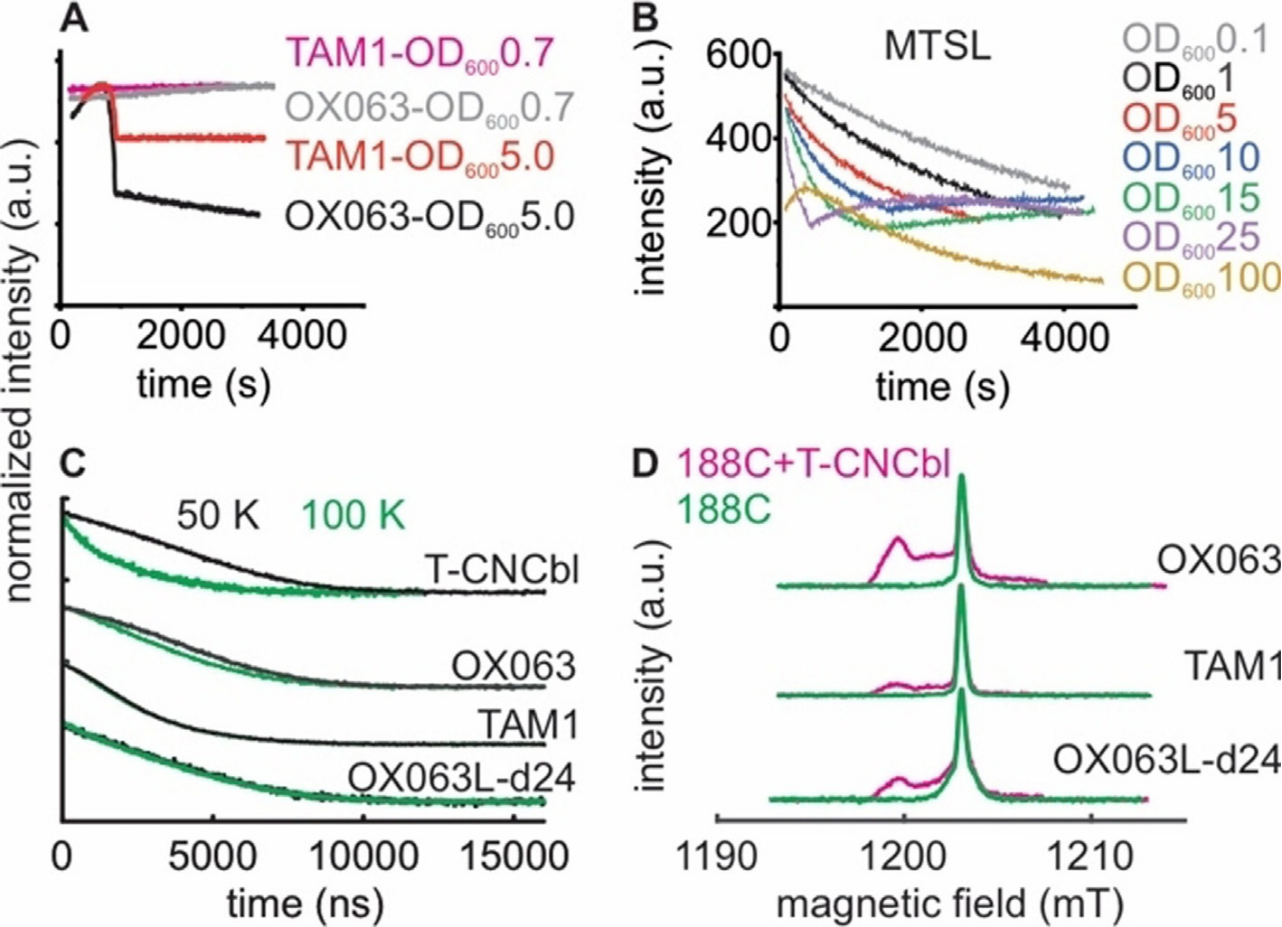
CW and pulsed ESR analysis of MTSL and trityl labels in *E. coli*. a) Stability curves for TAM1 and OX063 labels in *E. coli* suspension at OD_600_=5 or 0.7 as indicated. The signals are normalized for the maximum intensity. Due to a higher tendency for aggregation, the TAM1 concentration was always lower than the OX063 labels (see Figure S1, Supporting Information). The active resonator volume is ≈32 μL, which contains ≈2.5×10^6^ or ≈2.5×10^9^ cells at an OD_600_ of 0.1 or 100, respectively. b) Redox kinetics for MTSL in *E. coli* at different OD_600_ values as indicated. c) Transverse relaxation measurements (intensity normalized) for different trityls and T‐CNCbl bound to BtuB (as indicated) in *E. coli* at 50 or 100 K. d) Echo‐detected field‐swept spectra for the trityl labels attached to BtuB in *E. coli* at 100 K.

In a markedly different response, the stability of the nitroxides followed active redox kinetics with a fast reduction dominating the early part (Figure 3 b and Figure S2). The kinetics is highly dependent on the cell density, with the initial reduction getting faster at higher OD_600_ values. Also, the initial concentration detected after the deadtime (2 to 3 min) proportionately reduces with an increase in the cell density. Consequently, the kinetics at OD_600_=25 and 100 became noticeably different. The MTSL is very stable in the supernatant of the cell suspension and heat inactivation (to denature the proteins) of the cells considerably increased the label stability (Figure S3), which altogether reveal an active reduction of the MTSL by the cells. Interestingly, after this initial phase, the curves show an abrupt upward deflection especially at higher cell densities (OD_600_=10 to 25), revealing that the reverse oxidation gets prominent. Such a non‐exponential and biphasic kinetics imply that the reduction and oxidation process must be physically separated. The reduction might take place in the extracellular environment involving direct contact with the cell surface. Subsequently, those labels diffuse into the periplasm through the outer membrane porins, where they might get oxidized. The periplasm is a highly oxidizing environment and the rate of oxidation essentially gets limited by the diffusion across the OM and would be slow at the beginning. Despite this redox kinetics, we obtained a rather good labeling efficiency for BtuB. Therefore, it must be the case that the MTSL molecules attached to BtuB (or any other protein) are excluded from being reduced, which is in line with the involvement of an active molecule located on the cell surface in the reduction process (Figure S3).

With the BAM complex, we overexpressed the central BamA barrel in the commercial BL21(DE3) Rosetta2 cell lines and investigated the two positions which are located at the lateral gate. Available structures show that the BamA barrel exists in an inward‐open and a lateral‐open conformation.[^35^, ^36^] Based on the structures, in the inward‐open state, the investigated positions T434 (on the loop between β1and β2) and Q801 (on β16) are positioned within the membrane and would be least accessible to the spin labels (Figure 2 c). In the lateral open conformation, β‐sheets carrying 434 move away and bring the position out of the membrane plane with minimal change for 801. Labeling these single mutants with MTSL produced signals which are slightly larger than the WT background, showing that these positions are buried within the membrane as observed in the inward‐open structure. Interestingly, when both positions were mutated to cysteines, we obtained a very large signal. Thus, this double mutation appears to shift the conformational equilibrium in BamA, likely from the inward‐open to the lateral‐open conformation. Indeed, previous studies with purified BamA have shown that similar mutations at the lateral gate interface (434C + 807C) change the conformational equilibrium of the barrel.^[38]^ The PELDOR/DEER data for the double mutant gave an exponential decay devoid of an intramolecular interaction (Figure S4). This suggests that the double mutation might shift the equilibrium towards the lateral open conformation having least accessibility for 801C (Figure 2 c). The OX063L‐d24 label also gave a large signal for the double mutant (Figure 2 d). Overall, the signal is smaller than that obtained with MTSL, which might be due to the larger size of the OX063L‐d24 and proximity of the labeled positions with the membrane.

Although free trityls show a long *T_M_* in solution at ambient temperatures,^[24]^ attachment with proteins leads to a severe loss of *T_M_*.[^16^, ^19^, ^25^] When we labeled BtuB with TAM1 in membranes, the *T_M_* was reduced to ≈1.2 μs.^[8]^ Interestingly, in *E. coli* TAM1 gave a significantly longer *T_M_* close to ≈3.0 μs between 50–100 K (Table 1). The OX063 labels made an even larger difference giving a *T_M_* of ≈5.0 μs. The TEMPO‐labeled cyanocobalamin (T‐CNCbl) showed a comparable *T_M_* at 50 K, however, it was reduced to ≈2.0 μs at 100 K. Faster relaxation of trityls in the cellular environments was partly attributed to the endogenous manganese ions.^[27]^ In our case this might not be an issue as we expressed BtuB in the minimal media devoid of any manganese. The deuteriation of the OX063L‐d24 label did not prolong *T_M_*. However, it gave a broader FS, which makes it more suitable for trityl‐trityl PELDOR/DEER experiments (Figure 3 d and Figure S6). The longer linker and the interactions with the surrounding might account for the boarder FS.

Next, we performed PELDOR/DEER experiments on BtuB in *E. coli* following labeling with the three trityl labels. The distances were measured to the bound T‐CNCbl (30 μm) by observing either the trityls or the nitroxide (TEMPO) at their maxima. In the FS, these maxima are separated by ≈90 MHz. Compared to TAM1, the OX063 label has a similar linker whereas the OX063L‐d24 has a longer diester linker (Figure 1). Also, TAM1 is more lipophilic than OX063 labels. For observing the nitroxide we stayed at 50 K and moved to 100 K while observing the trityls. Observing the nitroxide consistently produced a lower modulation depth, revealing that the amount of added T‐CNCbl (30 μm) is above the trityl labeled BtuB available on the cell surface (4–8 μm, Table 1, Figure 4, and Figure S5). Observing the trityls gave a significantly larger modulation depth. TAM1 produced the largest modulation depth of ≈24 %, which is close to the maximum value achievable under the experimental settings (≈35 %). When observing the trityls at 100 K, the data could be acquired faster (2 ms shot repetition time), which together with a larger modulation depth and a favorable *T_M_* significantly reduced the acquisition time.

**Figure 4:**
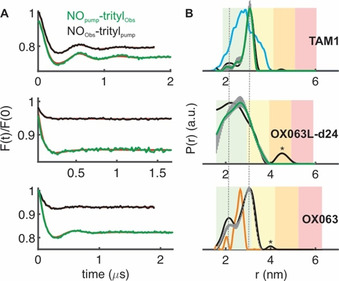
PELDOR/DEER spectroscopy of BtuB in *E. coli*. Distances were measured between position 188 and the spin labeled substrate (T‐CNCbl). BtuB was labeled with the three different trityls as indicated. a) Background corrected PELDOR/DEER data with the fits from Tikhonov regularization overlaid in red. b) The corresponding distance distributions. The reliability of the probability distribution is color coded using the longer trace as the reference. In the green zone, shape, width, and mean of the distribution are reliable, whereas in the yellow zone the width and the mean are reliable. The vertical lines indicate the total variation of the probability from a combined validation by changing the background and the noise level (see experimental procedures for details). For TAM1, the corresponding simulation (using MMM)^[39]^ is shown in cyan, and the distribution obtained after labeling with MTSL^[5]^ is shown in orange with the OX063 data. Asterix indicates artifact due to poor signal to noise.

Apparent differences are evident between the distance distributions obtained with the three trityl labels. Like MTSL, the OX063 gave a clearly bimodal distribution. With TAM1, the first component is significantly suppressed, and the second component gets narrower, whereas the distribution is smoothed with the OX063L‐d24 label. Simulation on the static structure (in the absence of a membrane bilayer) predicted a much broader distribution for TAM1 (Figure 4, cyan in the top panel). Having a very similar linker, the differences between TAM1 and OX063 must be due to a variation in the interaction of the label with its surroundings. It appears that with a more lipophilic core, the TAM1 interacts somewhat stronger than the hydrophilic OX063 labels, thereby producing a narrower distribution. Even with a much longer linker, OX063L‐d24 gave a relatively narrow distance distribution (2.5±0.5 nm). This observation further reveals that even for the OX063 labels, interaction with the surroundings might be significant enough to produce a narrow distance distribution. This is further evident from a relatively small difference in the overall distance distribution between the trityls and MTSL (in orange in the bottom panel), despite the trityls having a significantly larger size. Interestingly, with DNAs, it was shown that the linker length for the FTAM labels has no significant effect on the distance distribution due to the interaction with the terminal base pair.^[29]^ Although such interactions are not an issue for observing protein‐protein or protein‐ligand binding, simulation approaches that can explicitly take it into account are highly desired for probing conformational changes,.

Our results reveal that both FTAM and OX063 labels are very promising tools for the investigation of membrane proteins in *E. coli*. Despite having a larger size as compared to MTSL, trityls do not interfere with substrate binding. Comparison of the simulation for TAM1−T‐CNCbl distances with the experimental data (Figure 4 b, top panel) shows that the substrate binding and the conformation of the second loop are similar to the crystal structure (INQH). The observed discrepancy for the probability amplitudes might be largely accounted for by the interaction of the trityls with the surroundings, which could not be take into account during the simulation. Further supporting this notion, the above simulation shows an even better agreement with the OXO63 data, which also has a linker very similar to TAM1. For BamA, the low labeling efficiencies for the single cysteine mutants (434 and 801) directly correlate with the limited solvent accessibility as predicted for the corresponding structure in the membrane environment. Compared to MTSL, trityl labels are very stable in the cellular environment (Figure 3 a OD_600_0.7 and 3b OD_600_1.0). However, they show an aggregation tendency in a cell density‐dependent manner, which could be minimized or even eliminated at lower cell densities. The stability curves we observed are highly relevant for other in situ experiments using trityl or nitroxide labels, including the dynamic polarization (DNP)‐enhanced NMR spectroscopy.

In summary, a small background labeling, good labeling efficiency, long *T_M_*, and a narrow linewidth make trityls a very sensitive tag for the in situ experiments. These features allowed the observation of low micromolar BtuB‐T‐CNCbl complexes (≈4 μm) in *E. coli*, which would be impossible using nitroxide spin pairs. Orthogonal labeling as we demonstrated here would be extremely useful for studying membrane protein complexes such as BAM and the lipopolysaccharide transport (Lpt) system. They form multi‐subunit heterooligomeric complexes and orthogonal labels would allow the observation of conformational changes and inter‐subunit interactions using the same sample. For example, with the OX063L‐d24 sample, in addition to the trityl‐NO distance, we also probed the trityl‐trityl distances. In line with the NO‐NO[^5^, ^7^] and the trityl‐NO PELDOR data (Figure 4), this gave an exponential decay, revealing the absence of any BtuB‐BtuB interaction under our protein expression conditions (Figure S6). Thus, trityl as an orthogonal label provides not only high sensitivity but also high selectivity for the distance measurements. The OX063 label revealed a *T_M_* of ≈3.0 μs even at 200 K (Figure S7). Thus, with further optimizations, it would be feasible to observe key membrane protein complexes such as BAM and Lpt at physiological temperatures in *E. coli*.

## Conflict of interest

The authors declare no conflict of interest.

## Supporting information

As a service to our authors and readers, this journal provides supporting information supplied by the authors. Such materials are peer reviewed and may be re‐organized for online delivery, but are not copy‐edited or typeset. Technical support issues arising from supporting information (other than missing files) should be addressed to the authors.

Supplementary
