# Supplementary material for: In Situ Labeling and Distance Measurements of Membrane Proteins in E. coli Using Finland and OX063 Trityl Labels
Source: Chemistry. 2021 Jan 14;27(7):2299–304. doi: 10.1002/chem.202004606 (PMC7898545; doi:10.1002/chem.202004606)
Supplement: Supplementary file 1 — Supplementary [file CHEM-27-2299-s001.pdf]

Chemistry–A European Journal

Supporting Information

In Situ Labeling and Distance Measurements of Membrane Proteins in *E. coli* Using Finland and OX063 Trityl Labels

Sophie Ketter+,[a] Aathira Gopinath+,[a] Olga Rogozhnikova,[b] Dmitrii Trukhin,[b] Victor M. Tormyshev,[b] Elena G. Bagryanskaya,[b] and Benesh Joseph*[a]

Author Contributions

B.J. Conceptualization: Lead; Data curation: Lead; Formal analysis: Lead; Funding acquisition: Lead; Investigation: Supporting; Methodology: Lead; Project administration: Lead; Resources: Lead; Supervision: Lead; Writing – original draft: Lead; Writing – review & editing: Lead
S.K. Investigation: Equal; Writing – review & editing: Supporting
A.G. Investigation: Equal; Writing – review & editing: Supporting
O.R. Investigation: Supporting
D.T. Investigation: Supporting
V.T. Funding acquisition: Supporting; Investigation: Supporting; Methodology: Supporting; Supervision: Supporting; Writing – review & editing: Supporting
E.B. Conceptualization: Supporting; Funding acquisition: Supporting; Investigation: Supporting; Resources: Supporting; Supervision: Supporting; Writing – review & editing: Supporting.

Table of Contents

Table of Contents....1

Experimental Procedures....2

Cloning and Expression of BtuB and BamA....2

Spin labeling of BtuB and BamA using MTSL and trityls in *E. coli*....2

Sample preparation for the kinetic analysis of spin label stability....2

Continuous-wave EPR measurements....2

Pulsed EPR measurements....2

Figure S1. Kinetic analysis of the trityl labels in the *E. coli* suspension....4

Figure S2. Kinetic analysis of MTSL in the *E. coli* suspension....5

Figure S3. Kinetic analysis MTSL in the supernatant or with heat inactivated *E. coli* suspension....6

Figure S4. PELDOR/DEER data for BamA T434C and BamA T434C-Q801C mutants....7

Figure S5. PELDOR/DEER data for the BtuB T188C mutant in *E. coli*....8

Figure S6. Trityl-Trityl PELDOR/DEER measurement in *E. coli*....9

Figure S7. Phase memory time (T_M) measurement for BtuB188C labeled with OX063 at 200 K.....10

Author Contributions....10

References....10

Experimental Procedures

Cloning and Expression of BtuB and BamA

Cloning and protein expression of BtuB were performed as previously described.[1] The pAG1 plasmid encoding the wild-type or the 188C mutation in *btuB* was used for overexpression using the *E.coli* strain RK5016 (*argH, btuB, metE)*. Following inoculation with a 5 mL preculture (OD_{600} = 0.6 - 0.8), cells were grown for overnight in 2 L minimal media consisting of 0.6 M K_2HPO_4, 0.33 M KH_2PO_4, 0.08 M $(NH_4)_2SO_4$ and 0.02 M sodium citrate supplemented with 100 µg/mL ampicillin, 0.2 % w/v glucose, 150 µM thiamine, 0.01 % w/v methionine and arginine, 3 mM $MgSO_4$, and 300 µM $CaCl_2$. Subsequently, the flasks were transferred to 4 ºC. The *bamA* gene containing a N-terminal His_6 tag and a thrombin cleavage site after the signal sequence was custom synthesized (Geneart, Thermofisher Scientific) and subsequently cloned into the pCDFDuet-1 vector. Cysteines were introduced at the desired positions using the Q5 Site-directed mutagenesis kit (NewEngland Biolabs). BamA was expressed in *E. coli* Rossetta2(DE) cells.

Spin labeling of BtuB and BamA using MTSL and trityls in *E. coli*

Following overnight growth, cells were pelleted down and suspended in 45 mM MOPS buffer (pH 7.5) containing 55 mM NaCl (labeling buffer) to reach an OD_{600} value of 5.0 (1 mL total volume). The cells were labeled with 100 µM each of 1-oxyl2,2,5,5-tetramethyl-3-pyrroline-3-methyl methanethiosulfonate (MTSL, Toronto Research Chemicals, North York, ON, Canada) FTAM or the OX063 labels for 15 min at 25 °C. Following incubation cells were pelleted and washed two times using the same buffer. During each wash, the cells were gently and thoroughly mixed for several times and finally suspended into 25 µL of the buffer for the ESR experiments.

Sample preparation for the kinetic analysis of spin label stability

Following overnight growth, the cells overexpression BtuB WT protein were pelleted and suspended in the labeling buffer at appropriate dilutions to reach OD_{600} values of 0.1, 1, 5, 10, 15, 25, and 100. Subsequently, 100 µM MTSL was added, mixed thoroughly and gently and 20 µL cell suspension was transferred to the ESR tube. For the heat inactivation experiment, cells at OD_{600} of 5 were incubated at 65 °C for 15 min. The supernatant for the kinetic analysis was prepared by pelleting the cells at OD_{600} of 5. Subsequently, 100 µM MTSL was added, mixed thoroughly and 20 µL cell suspension was used for the ESR experiment. For the kinetic analysis of the trityls, 100 µM each of OX063, OX063L-d24, and TAM1 were added (in 3-4 equal portions) to cell suspensions at OD_{600} of 5. The suspension was thoroughly mixed and 50 µL sample was used for the ESR experiments.

Continuous-wave ESR measurements

Continuous-wave (CW) ESR measurements were performed at X-band frequency (9.4 GHz) on a Bruker EMXnano Benchtop Spectrometer. Nitroxide (MTSL) spectra were acquired with 100 kHz modulation frequency, 0.1 - 0.15 mT modulation amplitude, 0.6 - 1 mW microwave power, 1.28 - 5.12 ms time constant, 2.62 - 22.5 ms conversion time, and 18 mT sweep width. The TAM1, OX063, and the OX063L-d24 spectra were acquired at 100 kHz modulation frequency, 0.02 mT modulation amplitude, 0.6 - 1 mW microwave power, 1.28 ms time constant, 3.01 ms conversion time, and 2.5 - 18 mT sweep width. Measurements with a 10 kHz modulation frequency and a 0.05 G modulation amplitude produced an identical spectrum, ruling out any line distortion due to the experimental settings. All CW spectra were measured at room temperature as the first derivative of the absorption signal. The kinetic analysis using MTSL (in the supernatant and or heated cell suspension) or the trityl labels (in *E. coli* suspension) were performed at room temperature by recording the spectra with a 1.28 ms time constant and 2.62 ms conversion time with a delay of 2 s between the measurements. The OD_{600}-dependent kinetic analysis for MTSL was performed on a Bruker CW ESR E500 spectrometer equipped with a SHQE cavity. Spectra were acquired using 100 kHz modulation frequency, 0.1 mT modulation amplitude, 0.6 mW microwave power, 1.28 ms time constant, 5.12 ms conversion time, and 5.5 mT sweep width.

Pulsed ESR measurements

Following spin labeling, the cells were suspended into 25 µL of the labeling buffer. A 20% of deuterated glycerol (v/v) was always added, as well as 30 µM TEMPO-CNCbl (T-CNCbl) when required. The samples were transferred into 1.6 mm quartz ESR tubes (Suprasil, Wilmad LabGlass) and flash frozen in liquid nitrogen. Spectra were acquired at Q-band frequency (33.7 GHz) on a Bruker ELEXSYS E580 ESR spectrometer equipped with a PELDOR unit (E580-400U, Bruker), a continuous-flow helium cryostat (CF935, Oxford Instruments), a temperature control system (ITC 502, Oxford Instruments), an ELEXSYS SuperQ-FT accessory unit, an arbitrary waveform generator (SpinJet-AWG), a Bruker AmpQ 150 W TWT amplifier (Applied Systems Engineering Inc.), and a Bruker EN5107D2 dielectric resonator. Echo-detected field-swept experiments were performed at 100 K using a two pulse Hahn echo sequence $\pi/2 - \tau - \pi$ with 60 ns $\pi/2$ pulse and a 120 ns π pulse with an inter pulse delay (τ) of 400 ns. The echo was integrated for

350 ns and the shot repetition time was kept at 5 ms. Phase memory time (T_M) was determined using the same settings at 50 K and 100 K with a two-step phase cycling, while the τ was increased in 4 ns steps. For TAM1, OX063, and OX063L-d24, the shot repetition time (SRT) was kept at 20 ms and 5 ms at 50 K and 100 K, respectively. For the nitroxide, the SRT was set to 5 ms at 50 K and 2 ms at 100 K. The PELDOR/DEER measurements were performed with the dead-time free four-pulse sequence with a phase-cycled $\pi/2$ pulse. A 12 ns ELDOR pulse was used with a 16 ns and 32 ns $\pi/2$ and π observer pulses, respectively. While observing the nitroxide, the temperature was set to 50 K and increased to 100 K when observing the trityls. The frequency offset between the observer and pump pulses was 90 MHz and the shot repetition time was set to 2 ms. Trityl-trityl PELDOR/DEER using the OX063L-d24 labels were performed at 100 K. A 40 ns ELDOR pulse was used with a 25 ns $\pi/2$ and a 50 ns π pulse at 24 MHz offset. The data were analyzed using the MATLAB-based DeerAnalysis2019 software.[2] Briefly, the primary PELDOR/DEER data V(t)/V(0) were corrected for the intermolecular contribution (background) and the resulting form factors $F(t)/F(0)$ were fitted to distance distributions using a model-free Tikhonov regularization approach. For nitroxide labels, in the absence of free spins, the background fits into a stretched exponential decay with a two-dimensional spatial distribution of the spins (*d* = ~2.0) on the surface of *E. coli*.[5] This is also the case with BamA (see Fig. S4). For trityl labeled BtuB, a three-dimensional spatial distribution of the spins (*d* = ~3.0) fitted well for the background, which must be due to the presence of unbound T-CNCbl (see Fig. S5). For the trityl-trityl DEER, the data could not be fitted with *d* = ~2.0 and a three-dimensional spatial distribution of the spins (*d* = ~3.0) fitted the entire data into a monoexponential decay. Uncertainty in the probability distribution was calculated from a combined validation by changing the background (between 490-1440 ns for TAM1 and OX063 and 464-1138 ns for OX063L-d24 in 11 steps) and the L_{noise} level (to 1.5 in 10 steps) with a L_{prune} level of 1.15. PELDOR/DEER data simulations were performed using the MMM software.[3]

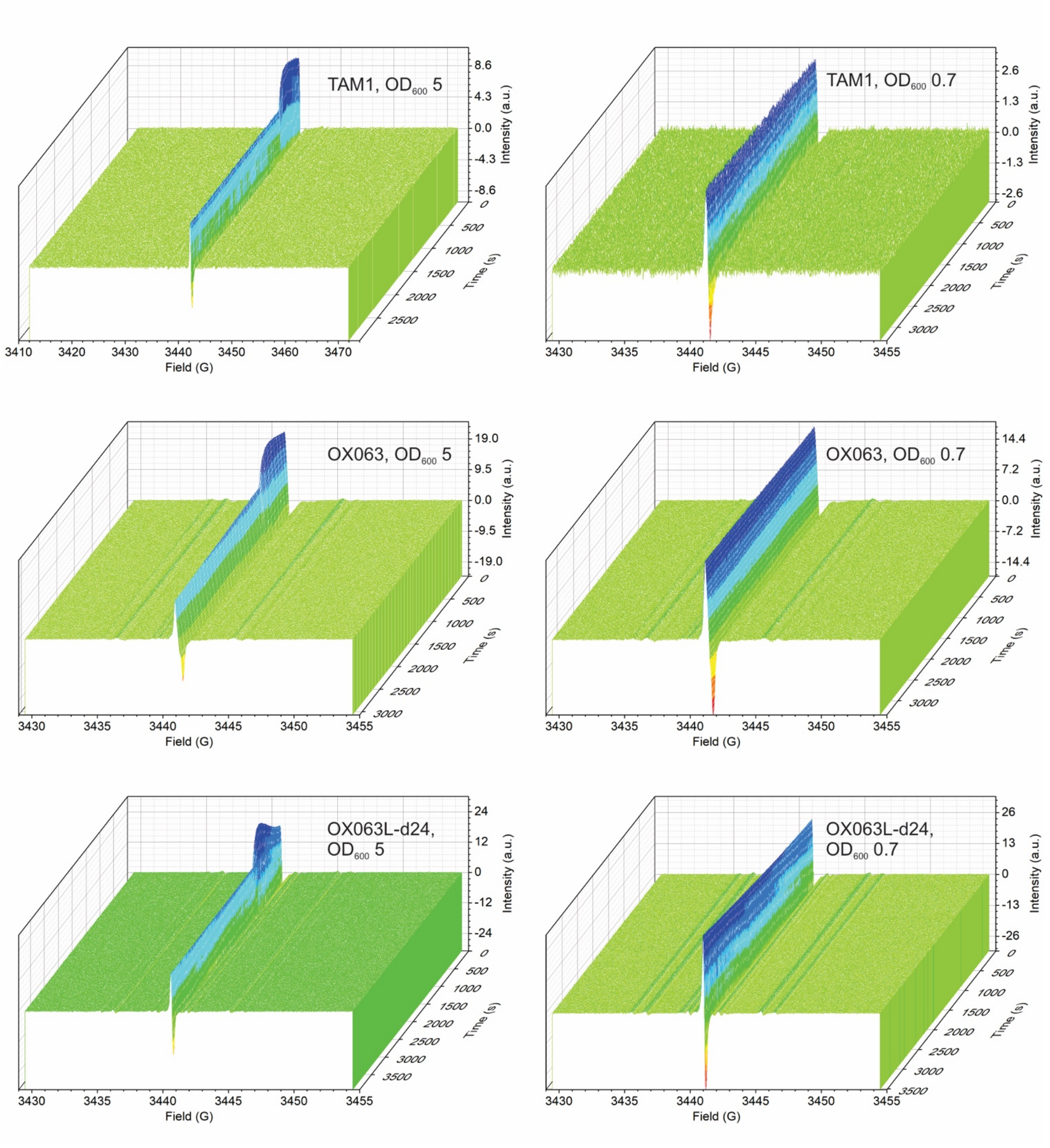

Figure S1. Kinetic analysis of the trityl labels in the *E. coli* suspension (OD_{600} of 5 or 0.7 with 100 μM spin label). The time-resolved spectra are shown with a color mapping (blue to red) along the y-axis to visualize the change in the signal intensity over time. The maxima of the signal intensity at OD_{600} of 5 were plotted against time to obtain the stability curves shown in Figure 3a. At OD_{600} of 0.7, all the labels revealed a stable behavior. Overall, TAM1 revealed a smaller signal due to the higher tendency for forming aggregates, which often sedimented to the bottom of the Eppendorf tube.

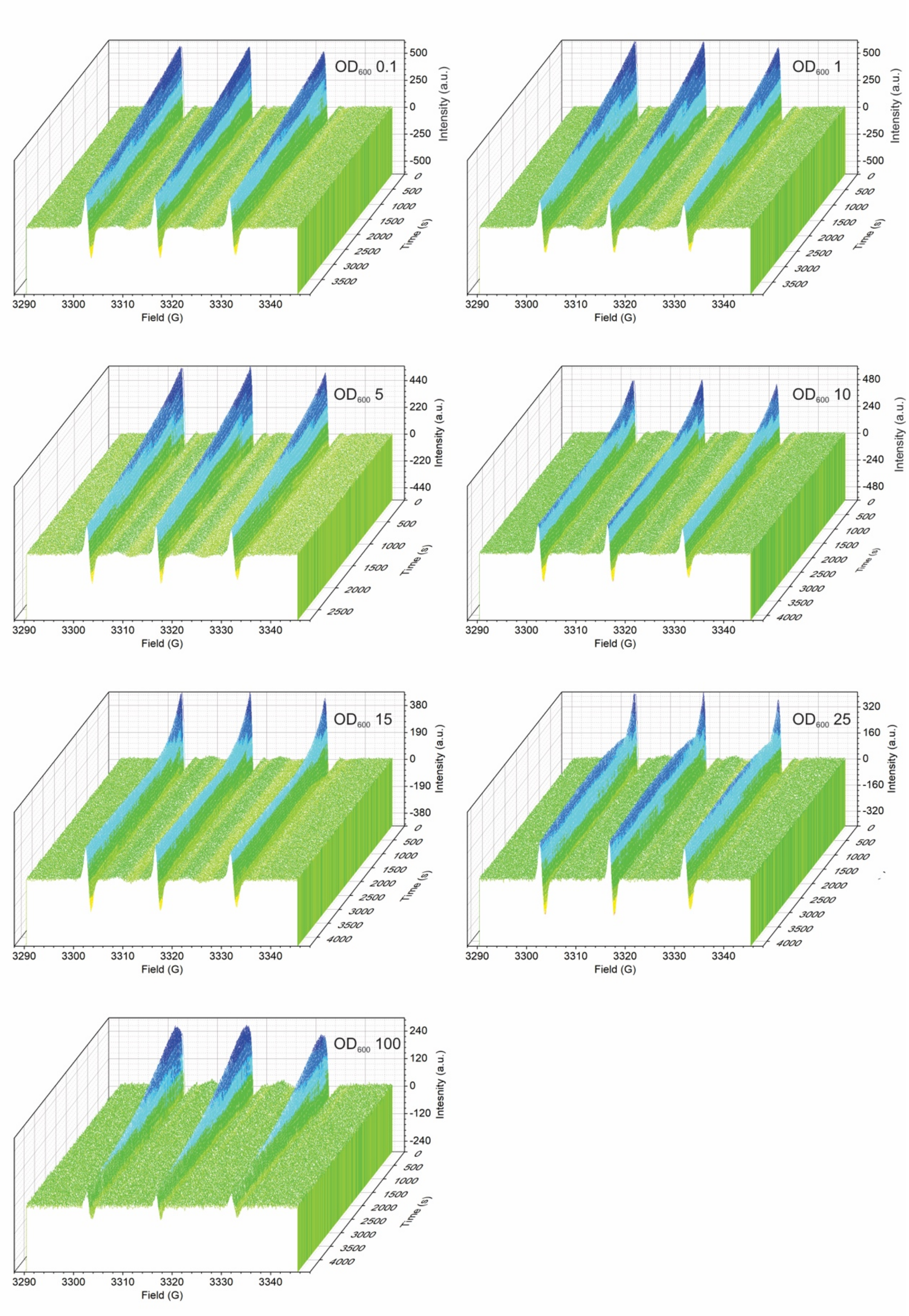

Figure S2. Kinetic analysis of MTSL in the *E. coli* suspension (100 μM spin label at different OD_{600} values as indicated). The time-resolved spectra are shown with a color mapping (blue to red) along the y-axis to visualize the change in the signal intensity over time. The maxima of the intensity for the central line were plotted against time to obtain the stability curves shown in Figure 3b.

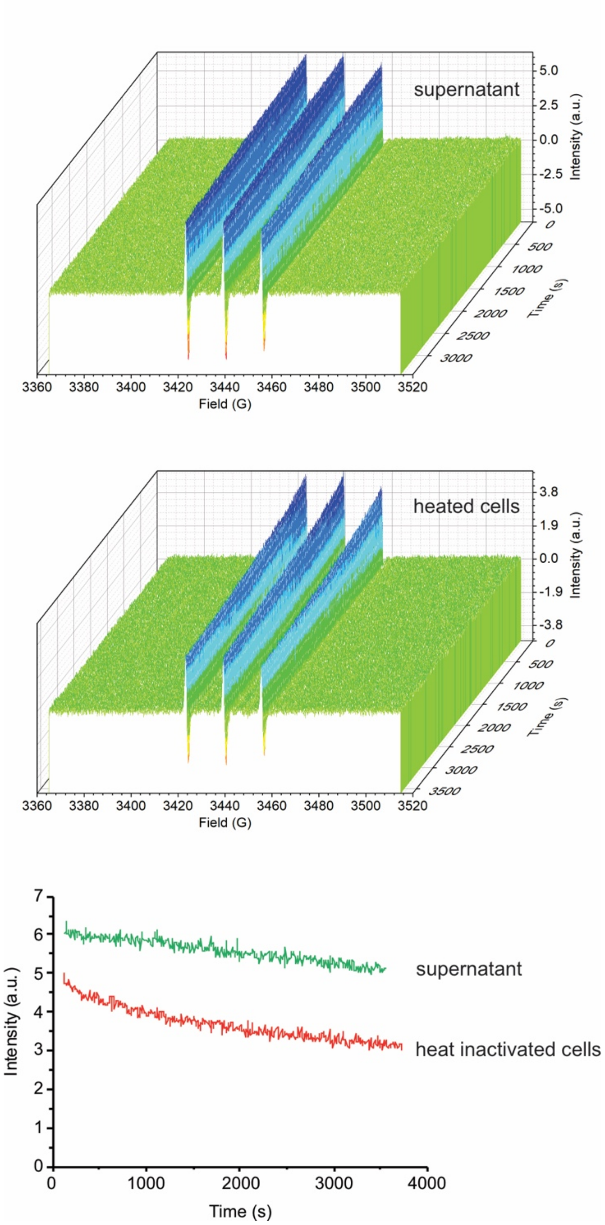

Figure S3. Kinetic analysis of MTSL in the supernatant or with heat inactivated (65 °C for 15 min) *E. coli* suspension (OD_{600} of 5 and 100 μM spin label). The time-resolved spectra are shown with a color mapping (blue to red) along the y-axis to visualize the change in the signal intensity over time. The maxima of the intensity were plotted against time to obtain the stability curves shown at the bottom. Compared to the *E. coli* suspension, the reduction is significantly reduced in the supernatant or upon heat inactivation.

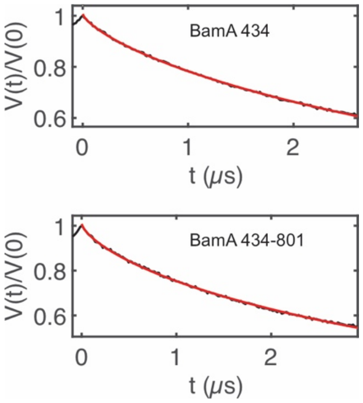

Figure S4. PELDOR/DEER data for BamA T434C and BamA T434C-Q801C mutants after labeling with MTSL in *E. coli*. The normalized experimental data V(t)/V(0) are shown in black, which fit into an exponential decay (overlaid in red) devoid of any characteristic distances for both the single and the double mutants. As we previously observed with BtuB,[4, 5] a dimensionality of *d* = ~2.0 fitted well for the spatial distribution of the spins, which must be due to the confinement of the protein in the membrane plane.

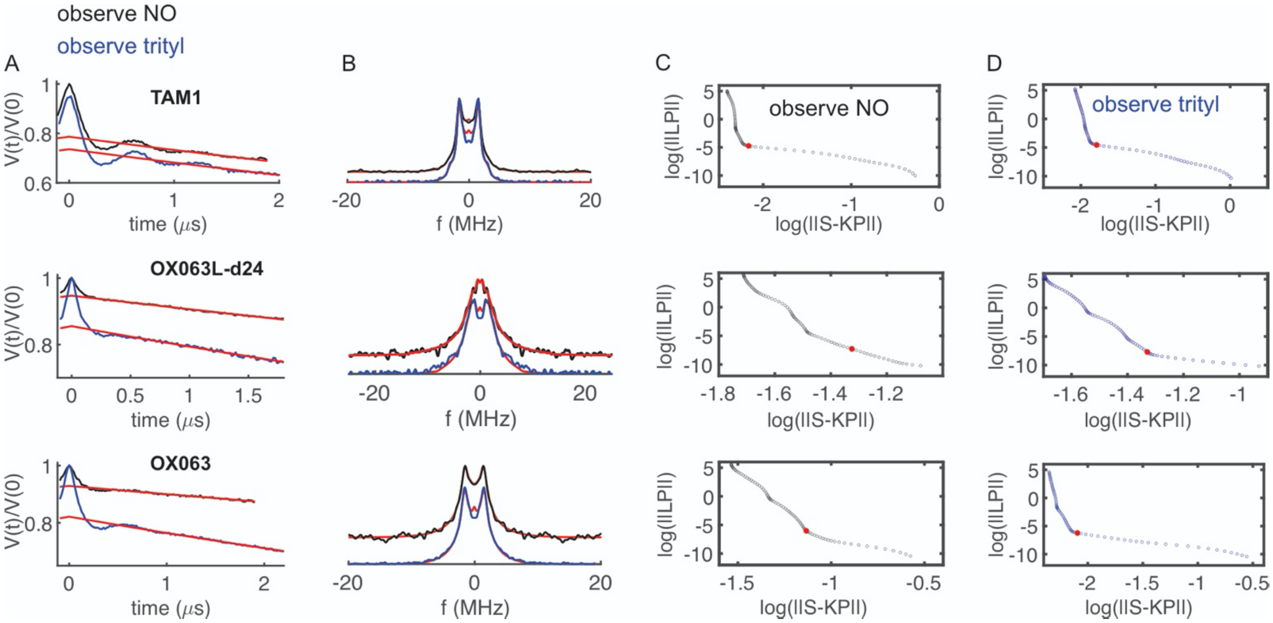

Figure S5. PELDOR/DEER data for the BtuB T188C mutant in presence of the spin-labeled substrate (T-CNCbl) in *E. coli*. Position 188C was labeled using the FTAM or the OX063 trityls as indicated. While observing the nitroxide, experiments were performed at 50 K (black) and for observing trityls, temperature was set to 100 K (blue). a) The normalized experimental data V(t)/V(0) with an exponential decay arising from intermolecular interactions overlaid in red. The latter showed a monoexponential decay (with a dimensionality of spin distribution, d = ~3.0), which must be due to an excess of the unbound T-CNCbl (30 μM) in the cell suspension. b) The corresponding dipolar spectrum with the fit from Tikhonov regularization overlaid in red. c, d) The L-curves from Tikhonov regularization with the chosen regularization parameter highlighted in red. The suggested regularization parameter was always used except for OXO63L-d24 when observing the nitroxide. Here, a regularization parameter that best matched the distribution while observing the trityl was manually chosen.

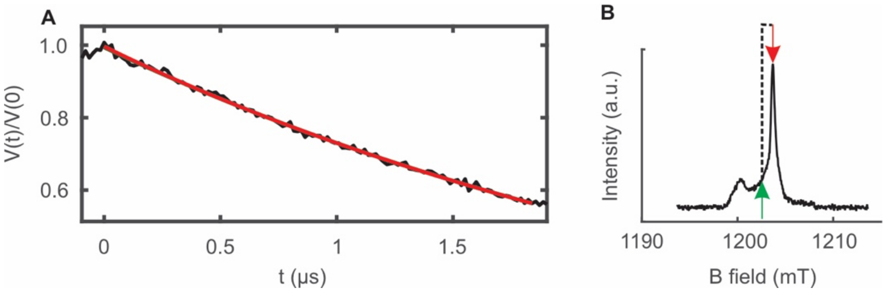

Figure S6. Trityl-Trityl PELDOR/DEER measurement in *E. coli*. BtuB T188C mutant was labeled with OX063L-d24 and PELDOR/DEER was performed (in presence of 30 μM T-CNCbl) at 100 K by pumping at the maximum of the spectrum with the observer pulses set with a 24 MHz offset. A) Normalized experimental data V(t)/V(0) is shown in black, which fits into a monoexponential decay with a dimensionality of spin distribution, *d* = ~3.0 (overlaid in red), revealing the absence of any BtuB-BtuB interaction under the experimental conditions. B) The position of the pump and observer pulses are shown on the echo-detected field-swept spectrum with red and green arrows respectively.

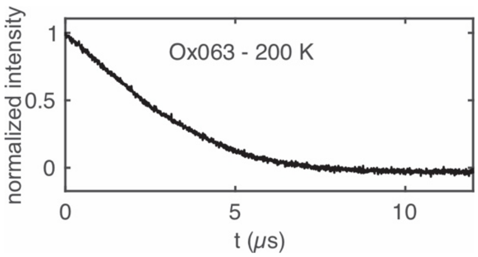

Figure S7. Phase memory time (T_M) measurement for BtuB188C labeled with OX063 at 200 K. Measurement was performed as described in the experimental section. The T_M was calculated as the time corresponding to the signal intensity at 1/e, which gave a T_M of ~3.0 μs.

Author Contributions

S.K. and A.G. made equal contributions. S.K. performed all the experiments with BtuB, A.G. performed all the experiments with BamA, O.R., D.T., and V.M.T. synthesized the trityl labels, S.K., A.G., and B.J. analyzed the data, E.G.B. and B.J. coordinated the project, and B.J. wrote the paper with inputs from all the authors.

References

[1] B. Joseph, A. Sikora, E. Bordignon, G. Jeschke, D. S. Cafiso, T. F. Prisner, *Angew. Chem. Int. Ed.* **2015**, *54*, 6196.
[2] G. Jeschke, V. Chechik, P. Ionita, A. Godt, H. Zimmermann, J. Banham, C. R. Timmel, D. Hilger, H. Jung, in *Appl. Magn. Reson. Vol. 30*, **2006**, pp. 473.
[3] Y. Polyhach, E. Bordignon, G. Jeschke, *Phys. Chem. Chem. Phys.* **2011**, *13*, 2356.
[4] B.Joseph, A.Sikora, E. Bordignon, G. Jeschke,D.S. Cafiso, T.F. Prisner, *Angew. Chem. Int. Ed.* **2015**, *54*, 6196
[5] B. Joseph, E. A. Jaumann, A. Sikora, K. Barth, T. F. Prisner, D. S. Cafiso, *Nat. Protoc.* **2019**, *14*, 2344.
